# How to build functional thylakoid membranes: from plastid transcription to protein complex assembly

**DOI:** 10.1007/s00425-012-1752-5

**Published:** 2012-09-14

**Authors:** Dagmar Lyska, Karin Meierhoff, Peter Westhoff

**Affiliations:** 1 Entwicklungs- und Molekularbiologie der Pflanzen, Heinrich-Heine-Universität Düsseldorf, Universitätsstr. 1, 40225 Düsseldorf, Germany; 2 Present Address: Physical Biosciences Division, Lawrence Berkeley National Laboratory and Department of Plant and Microbial Biology, University of California, Berkeley, CA 94720-3102 USA

**Keywords:** Chloroplast, Complex assembly, Endosymbiosis, Gene expression, Photosynthesis, Thylakoid membrane

## Abstract

Chloroplasts are the endosymbiotic descendants of cyanobacterium-like prokaryotes. Present genomes of plant and green algae chloroplasts (plastomes) contain ~100 genes mainly encoding for their transcription-/translation-machinery, subunits of the thylakoid membrane complexes (photosystems II and I, cytochrome *b*_6_ *f*, ATP synthase), and the large subunit of ribulose-1,5-bisphosphate carboxylase/oxygenase. Nevertheless, proteomic studies have identified several thousand proteins in chloroplasts indicating that the majority of the plastid proteome is not encoded by the plastome. Indeed, plastid and host cell genomes have been massively rearranged in the course of their co-evolution, mainly through gene loss, horizontal gene transfer from the cyanobacterium/chloroplast to the nucleus of the host cell, and the emergence of new nuclear genes. Besides structural components of thylakoid membrane complexes and other (enzymatic) complexes, the nucleus provides essential factors that are involved in a variety of processes inside the chloroplast, like gene expression (transcription, RNA-maturation and translation), complex assembly, and protein import. Here, we provide an overview on regulatory factors that have been described and characterized in the past years, putting emphasis on mechanisms regulating the expression and assembly of the photosynthetic thylakoid membrane complexes.

## Introduction

Chloroplasts are the characteristic organelles of plants and algae. They are the sites of photosynthesis, converting light to chemical energy and atmospheric CO_2_ to carbohydrates, as well as other essential processes like lipid metabolism, biosynthesis of amino acids, purine and pyrimidine bases, chlorophyll, and other tetrapyrroles (Neuhaus and Emes 2000; Finkemeier and Leister 2010).

The light-dependent reactions of photosynthesis, where light energy is fixed and converted to ATP and NADPH, are performed by four large thylakoid membrane-associated protein complexes: photosystems II and I (PSII and PSI) interconnected by the cytochrome *b*_6_ *f* complex, and ATP synthase.

Linear electron transport through the thylakoid membrane begins with the excitation of a chlorophyll pair, P680, at PSII and transfer of electrons to plastoquinone. P680^+^ is de-excited by electrons generated from water oxidation via the manganese cluster at the oxygen-evolving complex (OEC) at the lumenal side of PSII. Electrons from plastoquinol are transferred to PSI via the cytochrome *b*_6_ *f* complex and either plastocyanin or cytochrome *c*_6_. Finally, PSI passes electrons to ferredoxin, which reduces NADP^+^ to NADPH with the help of ferredoxin-NADPH oxidoreductase (FNR). Linear electron transport is coupled to the translocation of protons from the stroma to the lumen generating the proton motive force that drives ATP synthesis from ADP and inorganic phosphate by the ATP synthase (Nelson and Ben-Shem 2004). In addition, electrons generated on the reducing side of PSI can be re-injected into the plastoquinone pool, thus generating an additional proton motive force and boosting ATP synthesis, a process called cyclic electron transport (Munekage et al. 2004; Munekage and Shikanai 2005; Shikanai 2007).

Phylogenetic, structural, and biochemical analyses show that primary plastids developed 1.2–1.5 billion years ago from a cyanobacterium-like ancestor engulfed by a mitochondrium-possessing eucaryote (Martin and Russell 2003). Comparison of present cyanobacterial genomes with plastid genomes (plastomes) points to massive loss of genetic information as a consequence of endosymbiotic events. Whereas cyanobacteria like *Anabaena* sp. PCC 7120 and *Synechocystis* sp. PCC3168 have 5,366 and 3,268 protein-encoding genes, respectively (Kaneko et al. 1996, 2001), plastomes encode for significantly less proteins [*Arabidopsis thaliana*: 87 (Sato et al. 1999), *Chlamydomonas reinhardtii*: 99 (Maul et al. 2002)]. However, the total number of plastid proteins is estimated to be between 2,000 and 3,600 (Leister 2003; Richly and Leister 2004) and thus overall correlates to the protein content of cyanobacteria. These numbers imply that the majority of the genes were transferred to the nucleus or lost in the course of co-evolution of the symbiont and its host cell. Genes that were retained in the plastid genome mainly encode for its transcription- and translation-machinery, as well as for subunits of the thylakoid membrane complexes and the large subunit of ribulose-1,5-bisphosphate carboxylase/oxygenase (Martin et al. 2002; Timmis et al. 2004).

**Fig. 1:**
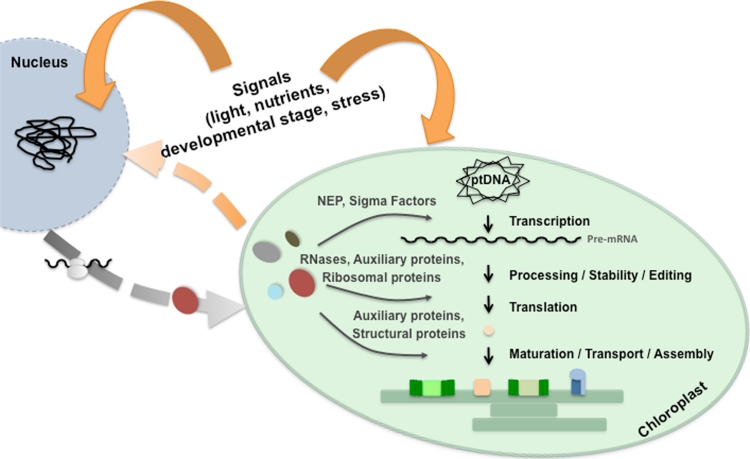
Overview of regulation levels of plastid gene expression and thylakoid membrane complex formation. Exo and endogenous signals influence nuclear and plastid gene expression, which again intercommunicate by retrograde and anterograde signals. Anterograde signals, i.e., nuclear-encoded factors that are synthesized in the cytosol and post-translationally transported into the chloroplast, control plastid gene expression from transcription, via maturation of transcripts, to translation. Also, the maturation, transport, and assembly of the thylakoid membrane complexes underlie regulation by nuclear-encoded factors. *ptDNA* plastid DNA, *NEP* nuclear-encoded RNA polymerase

The lateral transfer of major parts of the plastid ancestral genome to the nucleus and the emergence of novel nuclear-encoded, plastid-localized proteins require coordination of nuclear and plastid gene expression. Retrograde (plastid-to-nucleus) and anterograde (nucleus-to-plastid) signaling mechanisms evolved to permit communication between the two compartments (Woodson and Chory 2008). Plastids transmit their developmental or functional state by signals, which originate from (1) plastid gene expression, (2) pigment biosynthesis (e.g., tetrapyrroles), (3) reactive oxygen species, (4) redox states of the components of the photosynthetic electron transport, and (5) metabolite pool changes (Pogson et al. 2008; Kleine et al. 2009; Pfannschmidt 2010). On the other hand, the nucleus controls (1) all steps of plastid gene expression (transcription, RNA-processing, -editing, -stability, translation), (2) complex assembly, (3) protein import, and (4) enzyme activity in response to plastid, developmental and environmental signals (Fig. 1; Woodson and Chory 2008). In addition, it encodes structural components of the photosynthetic thylakoid membrane complexes and other complexes (Fig. 1; Herrmann et al. 1985; Wollman et al. 1999).

## Regulation of transcription

In higher plants, transcription of plastid genes is performed by distinct RNA polymerases, one plastid-encoded RNA polymerase (PEP) and two nuclear-encoded RNA polymerases (NEP) named RPOTp and RPOTmp (Maliga 1998; Liere and Börner 2007). The nuclear-encoded polymerases are monomeric phage-type enzymes (Lerbs-Mache 1993; Courtois et al. 2007; Swiatecka-Hagenbruch et al. 2007), which have evolved only in seed plants and mosses by duplication of the gene encoding a mitochondrial phage-type enzyme (Hedtke et al. 1997, 2000). These polymerases could not be identified in green algae. Most NEP-dependent promoters exhibit a YRTA sequence motif similar to plant mitochondrial promoters (Kühn et al. 2005) and show no similarity to PEP-dependent promoters. NEP is thought to drive expression of housekeeping genes during early phases of plastid and plant development (Lerbs-Mache 1993; Mullet 1993; Hajdukiewicz et al. 1997). However, NEP is also present in mature chloroplasts, driving transcription of genes encoding ClpP (a proteolytic subunit of ATP-dependent protease), ribosomal proteins and ribosomal RNA (Bligny et al. 2000; Cahoon et al. 2004; Azevedo et al. 2006; Swiatecka-Hagenbruch et al. 2008).

The plastid-encoded RNA polymerase is a multimeric eubacterial-type enzyme, which recognizes “consensus” promoters having the conserved sequences TTGACA and TATAAT centered at 35 and 10 bp upstream from a transcriptional initiation site, respectively (Harley and Reynolds 1987; Ishihama 1988; Lonetto et al. 1992). The PEP core enzyme consists of subunits α, β, β′ and β′′, which are encoded by the genes *rpoA*, *rpoB,* *rpoC1*, and *rpoC2* of the plastid genome, respectively (Shiina et al. 2005). PEP is regulated by nuclear-encoded sigma factors, which bind to the core enzyme to form the holoenzyme and initiate transcription. Most higher plant genomes encode six sigma factors (SIG1-6; except poplar, which has nine). In contrast, only one sigma factor gene has been identified in green algae, and it is not an ortholog to any plant gene (Carter et al. 2004; Lysenko 2007). Sigma factors are thought to have distinct functions in the regulation of plastid gene expression (Lysenko 2007; Lerbs-Mache 2011). Analyses of sigma factor mutants, mostly carried out in *Arabidopsis*, revealed that at least two sigma factors are essential for photoautotrophic growth (SIG2 and SIG6; Shirano et al. 2000; Ishizaki et al. 2005; Loschelder et al. 2006) and that each sigma factor is specific for one or several plastid genes (Lerbs-Mache 2011). Transcription of sigma factor genes *SIG1* and *SIG5* is induced by red and blue light, thus indicating a function under specific light conditions (Onda et al. 2008). Also, activity of sigma factors is believed to be modulated by redox reactions, phosphorylation, interaction with other proteins, and eventually proteolytic cleavage (Lerbs-Mache 2011). Several proteomic studies have identified additional putative components of the PEP complex of *Arabidopsis*, tobacco, and mustard (Suzuki et al. 2004; Pfalz et al. 2006; Schröter et al. 2010; Steiner et al. 2011). However, which of the identified factors truly associate with PEP complexes and their precise functions in transcription are still under discussion.

Originally, PEP-dependent transcription was assumed to play a role in later developmental stages of plants through transcription of photosynthesis-related genes (Mullet 1993). However, recent studies revealed that PEP is already active in seeds and is required for efficient germination (Demarsy et al. 2006). Also, both NEP and PEP were found to be active throughout the entirety of leaf development (Zoschke et al. 2007) and almost all plastid genes are transcribed from several independent promoters, allowing access to NEP as well as to PEP (Swiatecka-Hagenbruch et al. 2007). Together with the redundancy of some sigma factors and the lack of NEP and diversity of sigma factors in green algae, these results raise the question whether all of these factors are truly required for regulation of gene expression (Lerbs-Mache 2011). Maier et al. (2008) provide an alternative hypothesis, which proposes that the large number of regulatory nuclear-encoded factors has evolved in order to suppress point mutations occurring in plastid promoter regions, i.e., to assure constant plastid genome transcription by continually creating new promoter/polymerase pairs.

## Transcript maturation

Transcript maturation plays a vital role in the regulation of plastid gene expression. A broad range of nuclear-encoded factors has been described as performing and assisting in processing, editing, and splicing of plastid transcripts (reviewed in Stern et al. 2010; Barkan 2011). Recently, a proteomic study of nucleoids isolated from proplastids and mature chloroplasts from maize leaves identified a large number of proteins with RNA-related functions (1) indicating that transcript maturation occurs co-transcriptionally and (2) providing a set of new, so far uncharacterized proteins putatively involved in transcript maturation (Majeran et al. 2012). Here, we give a short overview of regulation of plastid transcript maturation. A detailed review on transcript processing is provided elsewhere in this issue.

### Transcript processing

In plastids, newly synthesized transcripts undergo several maturation steps prior to translation. Most genes are arranged in polycistronic gene clusters and transcribed from a single promoter (Herrmann et al. 1992; Sugiura 1992). As translation of monocistronic RNAs is often more effective than translation of polycistronic forms (Barkan et al. 1994; Sturm et al. 1994; Hirose and Sugiura 1997), intercistronic cleavage by endonucleases is required. Subsequently, 5′ and 3′ ends of cleaved products and of a priori monocistronic transcripts are modified by exonucleolytic and/or endonucleolytic trimming and polyadenylation. All these processes are dependent on nuclear-encoded proteins. Intercistronic cleavage is performed by endoribonucleases, which usually exhibit low sequence specificities, thus being supposed to be regulated by sequence-specific factors. RNase E (Schein et al. 2008) and RNase J (Li de la Sierra-Gallay et al. 2008) are candidates for endonucleases involved in intercistronic cleavage of plastid transcripts. A PPR (PentatricoPeptide Repeat) family protein, CRR2 from *Arabidopsis*, also exhibits endoribonuclease activity in vitro (Okuda et al. 2009) but is specific to the *rps7*-*ndhB* intergenic region (Hashimoto et al. 2003).

So far, 5′ end formation of transcripts was believed to be accomplished via site-specific intercistronic cleavage by an endonuclease since no protein with 5′ → 3′ exoribonuclease activity was known to be located in plastids. On the contrary, 3′ ends are supposed to be generated by the 3′ → 5′ exoribonuclease activity of polynucleotide phosphorylase (PNPase). This enzyme is sensitive to secondary structures and arrests at 3′ terminal stem–loop structures (Yehudai-Resheff et al. 2001). Recently, however a 5′ → 3′ exoribonuclease function was found for RNase J (Mathy et al. 2007) implying similar mechanisms for 3′ and 5′ end maturation. Some termini located in intergenic regions, e.g., *atpI*-*atpH* and *petB*-*petD* overlap after processing (Barkan et al. 1994; Pfalz et al. 2009) pointing to a role of 5′ → 3′ exonucleolytic degradation of 5′ ends. Pfalz et al. (2009) and Prikryl et al. (2011) postulate that endonucleolytic cleavages in intergenic regions occur stochastically and sequences are trimmed exonucleolytically until a prescribed position is reached. This position is either denoted by the presence of a stem–loop structure or it is marked by specific RNA-binding proteins. In maize, the PPR10 protein was found to define 5′ and 3′ ends of processed *psaJ*-*rpl33* or *atpI*-*atpH* intergenic regions, respectively (Pfalz et al. 2009). During binding to the 5′ end of *atpH*, PPR10 also activates translation by releasing a ribosome-binding region from an RNA-duplex (Prikryl et al. 2011). Similar functions are suggested for other PPR- and TPR-like proteins specific for a diverse set of transcripts (Barkan 2011). The discovery of a set of small RNAs (sRNAs) accumulating in chloroplasts is consistent with a role of different proteins serving as “caps” for processed transcripts. These sRNAs are believed to be the binding sites of capping proteins since their positions within transcripts overall match the 5′ and 3′ ends of processed transcripts (Ruwe and Schmitz-Linneweber 2012; Zhelyazkova et al. 2012).

### RNA splicing

Many plastid transcripts (mRNAs and tRNAs) harbor introns, which must be removed prior to translation. Depending on splicing mechanisms and primary and secondary structures, organellar introns are classified into group I and group II introns, respectively (Houghland et al. 2006; Pyle and Lambowitz 2006; Fedorova and Zingler 2007). Group II introns are sub-divided into group IIA to group IID, with only group IIA and IIB occurring in flowering plants (Michel et al. 1989).

Higher plant plastids exhibit only one group I intron located in the *trnL* transcript, whereas there are five in *C. reinhardtii* and even more in other *Chlamydomonas* species (Turmel et al. 1993). However, there is a set of about 20 group II introns conserved among higher plant organellar genomes (Schmitz-Linneweber and Barkan 2007). Originally, introns were mobile genetic elements encoding a maturase, which facilitates splicing of the host intron (Eickbush 1999; Wank et al. 1999). This ability has been widely lost in higher plants. The only remaining intron-encoded maturase, MatK, is encoded by the *trnK* transcript and was found to co-immunopurify with all group IIA introns except *clpP* intron 2 (Zoschke et al. 2010). In fact, splicing of plastid introns is rather mediated by at least 12 nuclear-encoded proteins (de Longevialle et al. 2010).

Two main protein families were found in this context: CRM (Chloroplast RNA splicing and ribosome maturation; Barkan et al. 2007) domain proteins, which target introns from several genes (Stern et al. 2010), and PPR proteins (Schmitz-Linneweber and Small 2008) that seem to be generally specific to only one transcript (Stern et al. 2010). In addition, some proteins harboring different sequence motifs are involved in splicing.

However, despite the large number of splicing factors identified, there is no concrete mechanistic model for their function. It is hypothesized that one or more factors interact with introns in a sequence-specific manner and change their folding, thus enabling recognition by general splicing factors (de Longevialle et al. 2010). Whether this is the general mechanism of plastid intron splicing needs to be further elucidated.

### RNA editing

Editing is the process of modification of transcripts by altering individual nucleotides. It is a common mechanism in all plants, except liverworts (Freyer et al. 1997) and it is absent in algae and cyanobacteria. In general, cytidine (C) is exchanged to uridine (U) in chloroplasts, but in some hornworts and ferns U is converted into C (Kugita et al. 2003; Wolf et al. 2004). There are typically about 30 editing sites in plastids of vascular plants, e.g., *Arabidopsis* has 34 editing sites in 18 different genes (Tillich et al. 2005; Chateigner-Boutin and Small 2007). Editing often restores conserved amino acids required for protein function (Zito et al. 1997; Sugita et al. 2006) or creates new translation initiation codons (Hoch et al. 1991; Kudla et al. 1992; Neckermann et al. 1994).

The mechanism of editing is so far unknown. However, both, *cis*- and *trans*-acting elements have been proposed to play a role. On the *cis*-acting side sequence elements immediately upstream of the respective site are necessary, putatively serving as binding sites for *trans*-acting elements (Bock et al. 1996; Chaudhuri and Maliga 1996; Sasaki et al. 2006). Indeed, several exclusively nuclear-encoded *trans*-acting factors have been identified and all except one belong to the family of PPR proteins. CRR4, the first *trans*-acting factor to be identified, is required for editing of a C at position 2 in *ndhD* transcripts and thus restoration of an AUG start codon (Kotera et al. 2005). It is the only factor that has been demonstrated to directly bind to its target editing site (Okuda et al. 2006, 2007). Like CCR4, the majority of *trans*-acting PPR proteins are specific to one transcript (Okuda et al. 2007; Zhou et al. 2009; Cai et al. 2009). However, five PPR proteins have been reported to target up to three editing sites, suggesting that editing factors must be able to distinguish pyrimidines from purines and sometimes even recognize specific bases (Hammani et al. 2009).

## Regulation of translation

Translation of plastid transcripts is performed by bacterial-type 70S ribosomes, in line with the prokaryotic origin of chloroplasts. Although there is a certain degree of conservation among translation factors and most ribosomal proteins, bacterial and plastid translation machineries differ (Beligni et al. 2004), e.g., by lack or divergence of some ribosomal proteins and gain of protein components for translational regulation of plastid gene expression (Zerges 2000; Beligni et al. 2004). Thus, both the large and the small ribosomal subunit also include nuclear-encoded plastid-specific ribosomal proteins (PSRP), which were characterized in spinach, *Arabidopsis* and *Chlamydomonas* plastids (Subramanian 1993; Yamaguchi et al. 2002, 2003; Tiller et al. 2012). Also, several nuclear-encoded proteins from *Chlamydomonas* and higher plants have been identified as transcript-specific translational regulators. The function of some of these factors will be discussed later.

### *Cis*-acting elements in plastid transcripts

Besides varying *trans*-acting factors, the organization of *cis*-elements displays a major difference between plastid and bacterial translation. In bacteria, translation is initiated by binding of the 3′ terminus of the 16S rRNA to the Shine–Dalgarno (SD) sequence (typically GGAGG) in the 5′ UTR of the transcript. The SD sequence is located between 5 and 9 nucleotides upstream from the start codon and mediates correct positioning of the ribosome for translational initiation (Laursen et al. 2005; Yusupova et al. 2001). SD-like sequences can also be found in 5′ UTRs of plastid transcripts, but they differ in position and sequence (Sugiura et al. 1998). While translation of some transcripts is dependent on SD-like sequences (*rbcL*, *atpE*, and *rps14* from tobacco; *psbA* from *Chlamydomonas*), other transcripts are only partially dependent (*rps12* and *petB* from tobacco) or independent of them (*psbA* and *atpB* from tobacco; *petD*, *atpB*, *atpE*, *rps4*, and *rps7* from *Chlamydomonas*; Mayfield et al. 1994; Sakamoto et al. 1994; Hirose and Sugiura 1996; Fargo et al. 1998; Hirose and Sugiura 2004a, b). Interestingly, the *rps2* SD-like sequence from tobacco was even shown to be a negative regulatory element for translation (Plader and Sugiura 2003). The dispensability of SD-like sequences in 5′ UTRs of plastid transcripts points to the existence of distinct regulatory *cis*-elements.

As an example, the 5′ UTR of the *psbA* transcript in tobacco possesses a SD-like sequence located at −33 nt upstream of the start codon. In tobacco this element is dispensable for translation (Hirose and Sugiura 1996), which stands in contrast to the requirement of a SD-like sequence in *Chlamydomonas psbA*-translation (Mayfield et al. 1994). Indeed, three other elements within the 5′ UTR of tobacco *psbA* have been identified: two sequences complementary to the 3′ terminus of chloroplast 16S rRNA (RBS1 and RBS2) located at −9 and −22 nt upstream of the start codon, respectively and an AU rich sequence (UAAAUAAA) located between RBS1 and RBS2 (Hirose and Sugiura 1996). It is hypothesized that RBS1 and RBS2 interact cooperatively with the 3′ end of 16S rRNA resulting in looping out of the AU rich sequence, which facilitates the interaction of *trans*-acting factors (Hirose and Sugiura 1996). Similar sequence elements have been described for the 5′ UTR of *psbD* in *Chlamydomonas*, with a SD-like motif (PRB1) and the PRB2 site at −13 and −30 nt upstream of the start codon, respectively and a polyU-rich element located in between (Nickelsen et al. 1999). Like the sequence elements from the *psbA* 5′ UTR, those of *psbD* are thought to serve as binding sites for *trans*-acting factors (Nickelsen et al. 1999; Ossenbühl and Nickelsen 2000). Other *cis*-elements affecting protein synthesis were found in the 5′ UTRs of *psbC*, *petD* and *rps*7 of *Chlamydomonas* (Zerges et al. 1997, 2003; Fargo et al. 1999; Higgs et al. 1999) and the *atpB* mRNA of tobacco (Hirose and Suigura 2004b) mainly in the context of targets for *trans*-acting factors.

### *Trans*-acting translational regulators

*Trans*-acting factors of translation are encoded in the nucleus and are generally specific to single transcripts. As mentioned above, many factors that mediate transcript stability by binding to the 5′UTRs of their target transcripts are also proposed to assist in translation initiation by releasing ribosome-binding sites (Barkan 2011; Prikryl et al. 2011). These functions have been assigned to many PPR proteins like the above-mentioned PPR10, HCF152, CRP1, and PPR38 (for *atpI*-*atpH*, *psbH*-*petB*, *petB*-*petD*, and *clpP*-*rps12*, respectively) that were identified in *Arabidopsis*, maize and the moss *Physcomitrella patens* (Meierhoff et al. 2003; Schmitz-Linneweber et al. 2005; Hattori et al. 2007; Hattori and Sugita 2009; Pfalz et al. 2009; Barkan 2011; Prikryl et al. 2011) and for the HAT (Half-A-Tetratricopeptide repeat) protein HCF107 of *Arabidopsis* and maize that regulates *psbH* transcript stability and translation (Felder et al. 2001; Sane et al. 2005; Hammani et al. 2012).

Besides general regulation of plastid translation, many trans-factors were described as modulating translation in a light- and assembly-dependent manner (Marin-Navaro et al. 2007). The best-studied mechanism of light-regulated translation was described for the *psbA* transcript in *Chlamydomonas*. A stem–loop structure in the 5′ UTR (Mayfield et al. 1994) is bound by a complex of several proteins upon illumination. RB47 is a poly A-binding protein (cPAB1) whose binding to the *psbA* 5′UTR is enhanced under reducing conditions (Kim and Mayfield 1997; Yohn et al. 1998). Modulation of RNA-binding properties of cPAB1 in response to the redox status of the cell is believed to be regulated by RB60 and TBA1, a putative protein disulfide isomerase (cPDI) and oxidoreductase homolog, respectively (Alergand et al. 2006; Kim and Mayfield 1997; Somanchi et al. 2005). In addition, ADP-dependent phosphorylation of cPDI is presumed to control the RNA-binding capacity of cPAB1 (Danon and Mayfield 1994). Another protein, RB55, was identified as part of the complex bound to the *psbA* 5′ UTR, but it has not yet been cloned or characterized (Danon and Mayfield 1991). Finally, RB38, another RNA-binding protein, which functions independent of the redox status, was described to bind specifically to uridine-rich sequences of the *psbA* 5′UTR (Barnes et al. 2004). However, RB38 was found to be identical to RBP40 from *Chlamydomonas*, which is involved in *psbD*-rather than *psbA*-translation (Schwarz et al. 2007). RBP40 binds to the polyU-rich element of the *psbD* 5′UTR (Ossenbühl and Nickelsen 2000) and destabilizes the stem–loop structure thereby enabling binding of the small ribosomal subunit to the initiation codon (Klinkert et al. 2006). Guidance of RBP40 to its target sequence is mediated by the stability factor NAC2, which, together with RBP40, is part of a high molecular weight complex of approximately 550 kDa (Schwarz et al. 2007). As is the case for *psbA*, translation of *psbD* is induced by light (Malnoe et al. 1988). A recent study provided evidence for a dynamic formation of the NAC2/RBP40 complex, which is based on an intermolecular disulfide bridge that is reduced in the dark causing dissociation of RBP40 from the complex and preventing translation initiation (Schwarz et al. 2012).

Besides light regulation, plastid translation can also be modulated by the assembly status of proteins into their respective complexes. This regulatory process is referred to as “Control by Epistasy of Synthesis” (CES) and has been reported for all four thylakoid membrane complexes (Choquet et al. 1998; Wostrikoff et al. 2004; Minai et al. 2006; Drapier et al. 2007). Regulation of the cytochrome *f* subunit of the cytochrome *b*_*6*_ *f* complex from *Chlamydomonas* is the best-studied process so far. When not assembled into the complex, cytochrome *f* interacts with MCA1, which usually binds and stabilizes the *petA* transcript and interacts with the translational factor TCA1 (Raynaud et al. 2007; Boulouis et al. 2011). Interaction with unassembled cytochrome *f* induces proteolysis of MCA1. This destabilizes the *petA* transcript and prevents its translation, which finally results in autoinhibition of cytochrome *f* synthesis (Raynaud et al. 2007; Boulouis et al. 2011).

Regulation of translational initiation is clearly the most important step of plastid translation. However, elongation and termination also display targets for regulatory processes. Elongation of the D1 protein is regulated by light (Mühlbauer and Eichacker 1998; Zhang et al. 2000), the presence of co-factors (Mullet et al. 1990) and the availability of assembly partners (de Vitry et al. 1989). The nuclear-encoded factors VIR-115 from barley and AC115 from *Chlamydomonas* are assumed to stabilize translation intermediates of D1 or D2, respectively, and facilitate their folding, co-factor binding, and/or co-translational membrane insertion (Kim et al. 1994; Rattanachaikunsopon et al. 1999).

Recognition of stop codons and translational termination is dependent on release factors in both eukaryotes and prokaryotes. In bacteria, the release factors RF1 (prfA) decoding UAA and UAG and RF2 (prfB) decoding UAA and UGA have been identified (Scolnick et al. 1968; Nakamura and Ito 1998). In *Arabidopsis*, a homolog to prfB, AtPRFB1/HCF109, has been cloned and shown to be required for termination of transcripts with UGA and UAA stop codons and to regulate transcript stability and protein synthesis (Meurer et al. 2002).

## Post-translational regulation: protein maturation and complex assembly

The last important steps of plastid gene expression include protein maturation, i.e., co-factor binding and proteolytic processing, and the assembly of all subunits to form an active complex. In addition, thylakoid membrane proteins have to be inserted into the lipid bilayer or transported across it. All of these processes can occur post- or co-translationally and are dependent on both general and complex-specific auxiliary factors.

### Membrane insertion

Transport of complex subunits across or insertion into the thylakoid membrane is facilitated by four distinct pathways. Both the secretory (Sec) and twin-arginine-translocase (Tat) pathways transport proteins across the thylakoid membrane into the lumen. The “spontaneous” and the SRP pathways insert proteins into the membrane, the latter being specific to LHCP (light-harvesting chlorophyll binding) family proteins (Schünemann 2004; Luirink et al. 2005; Aldridge et al. 2009). Moreover, LHCP membrane insertion is dependent on the ALB3 translocase, which is the plastid homolog to the membrane insertion factors Oxa1p and YidC from mitochondria and *Escherichia coli*, respectively (Moore et al. 2000; Bellafiore et al. 2002; Kuhn et al. 2003). The SRP pathway is believed to recruit LHCPs from the stroma and target them to the membrane and ALB3 (Aldridge et al. 2009). ALB3 also interacts with the chloroplast SECY translocase (Klostermann et al. 2002; Pasch et al. 2005), which is believed to be involved in co-translational membrane insertion of the D1 protein (Zhang et al. 2001). Yeast split-ubiquitin and co-immunoprecipitation experiments revealed a direct interaction between ALB3 and subunits from PSII and PSI and the CF_0_III subunit from ATP synthase (Pasch et al. 2005; Göhre et al. 2006), indicating a general role of ALB3 in membrane insertion of proteins. The maturation and assembly of the thylakoid membrane complexes are likely mediated by more specific factors since each complex and each subunit exhibit different properties.

### Post-translational modifications/subunit maturation

Maturation of plastid proteins may also involve their proteolytic truncation. The D1 subunit of the PSII reaction center is the best-known example for this mode of protein modification. D1 is synthesized as a precursor (pD1) with a C-terminal extension of nine amino acid residues in higher plants (Marder et al. 1984; Takahashi et al. 1988). During its integration into the thylakoid membrane, the D1 precursor is processed to its mature form by the nuclear-encoded lumenal carboxy-terminal processing protease (CtpA; Anbudurai et al. 1994; Fujita et al. 1995). Recently, additional components facilitating the processing of the D1 precursor protein have been identified in *Synechocystis* (PratA; Klinkert et al. 2004) and *Arabidopsis* (LPA19; Wei et al. 2010), although their precise function is not clear yet. C-terminal processing was reported to be a prerequisite for binding of the manganese cluster to PSII (Nixon et al. 1992; Roose and Pakrasi 2004).

Another aspect of protein maturation is the binding of co-factors. The photosynthetic complexes contain pigments for light harvesting and charge separation as well as redox-active factors for electron transfer. PSII possesses several chlorophylls and xanthophylls, two pheophytins, two hemes, one manganese cluster, one bicarbonate, two Ca^2+^ ions, one Fe^2+^ ion, and one Cl^−^ ion (Guskov et al. 2009). Similarly, PSI is associated with a large number of chlorophylls and xanthophylls, in line with the function of both photosystems in light harvesting. Redox reactions are facilitated by two phylloquinones and three [4Fe–4S] clusters (Amunts et al. 2007). Finally, the cytochrome *b*_*6*_ *f* complex binds four hemes (two b- and c-type hemes each), one [2Fe–2S] cluster and one chlorophyll and xanthophyll each (Kurisu et al. 2003; Stroebel et al. 2003).

Plastid Fe–S clusters are assembled by machinery related to the eubacterial SUF (Sulfur Utilization Factor) system (Balk and Pilon 2011). Attachment of assembled clusters to their target proteins seems to require further components, such as scaffold and carrier/transfer proteins. So far, only two proteins have been identified that may play a role in [4Fe–4S] cluster binding to PSI: the *Arabidopsis* HCF101 protein and cyanobacterial RubA (Schöttler et al. 2011). Recombinant HCF101 was found to transiently bind [4Fe–4S] clusters and transfer them onto apoproteins (Schwenkert et al. 2010). Based on spectroscopic measurements using *Synechococcus* sp. mutant strains, RubA was hypothesized to be specifically required for incorporation of the [4Fe–4S] cluster associated with PsaAB (F_x_; Shen et al. 2002). Homologs of RubA are encoded by higher plant genomes but their function has not been elucidated so far.

As mentioned above, the cytochrome *b*_6_ *f* complex is associated with four hemes. The two non-covalently bound b-type hemes are bound by the cytochrome *b*_6_ polypeptide and are believed to bind spontaneously (Robertson et al. 1994). On the contrary, the two c-type hemes bind covalently to cytochrome *b*_6_ and cytochrome *f*, respectively.

For biogenesis of c-type cytochromes, four distinct pathways (system I–IV) have been characterized so far, although there is evidence for additional mechanisms (Allen et al. 2004; de Vitry 2011). System III, which occurs in mitochondria of fungi, vertebrates and invertebrates, is the simplest system and consists of a heme lyase as the single component (Dumont et al. 1987; Giegé et al. 2008). In contrast, systems I, II and IV are more complex. System I (or Ccm system) occurs in archaea, α- and γ-proteobacteria and the mitochondria of plants and red algae, and consists of up to 10 membrane-bound proteins (Allen et al. 2008; Sanders et al. 2010). System II operates in some gram-positive bacteria, cyanobacteria, some β-, δ- and ε-proteobacteria and plastids of plants and algae (Kranz et al. 2002). In plastids, it is required for the biogenesis of cytochromes *f* and *c*_6_ (Rurek 2008). Cytochrome *f* possesses a classical CXXCH heme-binding motif whose two cysteines form thioether linkages with the vinyl groups of the heme. The apoprotein is synthesized in the plastid stroma but targeted to the electropositive thylakoid lumen in a Sec-dependent way (Rohl and van Wijk 2001), where the conversion of apo- to holo-cytochrome *f* occurs. Ligation of heme to cytochrome *f* requires several steps. The heme is synthesized in the stroma, and needs to be transported to the lumen. There it must be transferred to or kept in its reduced state to assure that the vinyl groups are chemically active. Similarly, the cysteine residues of the heme-binding motif in the cytochrome must be reduced to form thioether bonds to the heme vinyl groups (Kranz et al. 2009). At least six factors are involved in cytochrome *f* (and *c*_6_) maturation in *Chlamydomonas*: the nuclear-encoded proteins CCS1–CCS5, and the plastid-encoded CcsA (Howe and Merchant 1992; Xie and Merchant 1996, 1998; Inoue et al. 1997). CcsA and CCS1 (also: CCSB) are supposed to form a complex and function in heme delivery and its ligation to the apoprotein (Dreyfuss et al. 2003; Hamel et al. 2003). CcsA contains a “WWD” motif (WGXϕWXWD, where ϕ is an aromatic residue) exposed to the lumen, which is also found in two system I components and is thought to transport heme into the thylakoid lumen and present it to the apocytochrome for ligation (Xie and Merchant 1996; Goldman et al. 1998; Rurek 2008; Kranz et al. 2009). CCS1, on the other hand, is supposed to chaperone reduced apocytochromes (Rurek 2008). HCF164, a thioredoxin-like protein from *Arabidopsis* was also found to be required for cytochrome *f* maturation (Lennartz et al. 2001) and recently, CCS5 was described as its homolog in *Chlamydomonas* (Gabilly et al. 2010). Also, the thiol disulfide transporter homolog CCDA is required for cytochrome *f* maturation in *Arabidopsis* but it has not yet been characterized in *Chlamydomonas* (Page et al. 2004). CCS5 and CCDA together with the recently discovered CCS4 protein seem to be involved in a thiooxidation/thioreduction pathway in which reducing equivalents are transferred to the electropositive side of the thylakoid membrane (lumen) and used for the reduction of apocytochromes (Rurek 2008; Gabilly et al. 2011).

In contrast to cytochrome *f*, cytochrome *b*_6_ binds its *c*-type heme (heme c_i_) on the electronegative, i.e., the stromal side of the thylakoid membrane, by only a single cysteine residue (Cys35; Kurisu et al. 2003; Stroebel et al. 2003). The maturation pathway mediating this atypical heme binding, the CCB system or system IV, requires at least four nuclear-encoded factors, CCB1–4, and is conserved among species performing oxygenic photosynthesis (Kuras et al. 2007; Lyska et al. 2007; Lezhneva et al. 2008; de Vitry 2011). Apparently, insertion of heme c_i_ to cytochrome *b*_6_ occurs after binding of the non-covalent *b*-type hemes and requires several steps in which different CCB-CCB and CCB-cytochrome *b*_6_ complexes are formed (Saint-Marcoux et al. 2009). Since heme synthesis and incorporation into apocytochrome *b*_6_ take place on the same side of the thylakoid membrane, no thioredox or translocation machinery is required and the CCB proteins are more likely to operate as a heme-chaperoning and -delivery system (de Vitry 2011). Conserved stroma-exposed tryptophan, tyrosine and/or phenylalanine residues in the CCB proteins that are expected to interact with heme (similar to the WWD motif in CcsA, see above) support this hypothesis (Kuras et al. 2007; de Vitry 2011). How the CCB proteins mediate heme attachment to apocytochrome *b*_6_ and whether there are more, so far undiscovered factors participating in these processes still needs to be elucidated in future studies.

### Assembly of complexes

Assembly of thylakoid membrane complexes is accomplished in a step-by-step manner. Usually, one protein serves as an anchor or scaffold for subsequent assembly steps. For PSII, a complex consisting of the D2 protein and the α and β subunits of cytochrome *b*_559_ seems to be the initiation point of assembly (Komenda et al. 2004; Rokka et al. 2005; Minai et al. 2006). Subsequently, the D1 and PsbI proteins are attached to form the reaction center (RCII; Rokka et al. 2005; Minai et al. 2006; Dobáková et al. 2007). The addition of the inner antenna protein CP47 leads to the formation of the RC47 complex and facilitates binding of several small subunits like the phosphoprotein PsbH (Komenda et al. 2004; Rokka et al. 2005; Minai et al. 2006). After incorporation of CP43 the monomeric core complex is complete and assembly of the manganese cluster and oxygen-evolving complex can occur, as well as dimerization and supercomplex formation (Rokka et al. 2005). A multitude of assembly-assisting proteins has been identified for PSII, although their mode of action is largely unknown. LPA1 from *Arabidopsis* is an intrinsic membrane protein and is thought to function as a chaperone for PSII assembly by interaction with D1 (Peng et al. 2006). Another protein, PAM68 is assumed to associate with LPA1 and D1 in an early intermediate complex (Armbruster et al. 2010). Folding and/or assembly of CP43 into PSII depends on the ALB3 interacting proteins LPA2 and LPA3 (Ma et al. 2007; Cai et al. 2010). Similarly, HCF136 is involved in a very early assembly step of PSII since in absence of this factor, PSII subunits are synthesized but no stable complexes can be detected (Meurer et al. 1998; Plücken et al. 2002). At the level of supercomplex formation AtFKBP20-2, a lumenal immunophilin, and Psb29 were reported to play a role (Keren et al. 2005; Lima et al. 2006).

PSII is not only synthesized de novo, but it is constantly renewed due to photooxidative damage (Aro et al. 1993). The D1 protein is the major target of the PSII repair cycle (Mattoo et al. 1981; Ohad et al. 1990). Damaged PSII complexes are phosphorylated and migrate from the grana to the stoma lamellae, where D1 is degraded and replaced by a newly synthesized protein (Mulo et al. 2008). Degradation is predominantly mediated by FtsH and Deg proteases that act in a cooperative manner (Kato and Sakamoto 2009; Kato et al. 2012) and apparently the lumenal AtTLP18.3 protein is involved in degradation of D1, but also in dimerization of PSII reaction centers (Sirpiö et al. 2007). Chlorophyll released during repair may be bound by ELIP proteins in higher plants, which belong to the LIL (light-harvesting-like) protein family (Hutin et al. 2003). Reassembly of PSII is assumed to require the abovementioned general assembly factors in addition to repair-specific factors, like Hsp70 proteins and PPL1 (Ishihara et al. 2007; Mulo et al. 2008).

In contrast to the large number of auxiliary factors for assembly of PSII, only a few proteins have been identified in conjunction with PSI and the cytochrome *b*_6_ *f* complex. The starting point for cytochrome *b*_6_ *f* complex assembly is cytochrome *b*_6_, which rapidly interacts with subunit IV. This dimer is a prerequisite for cytochrome *f* binding and subsequent assembly of the small subunits PetG, PetL, PetM, and PetN. The Rieske protein appears to be bound only loosely to the complex and is likely to contribute to dimer stability, which is the active form of the cytochrome *b*_6_ *f* complex (Choquet and Vallon 2000; Kurisu et al. 2003; Stroebel et al. 2003). Biogenesis factors have only been identified in the context of (1) processing and/or stabilization of transcripts, (2) CES autoregulation of cytochrome *f*, and (3) co-factor binding, which is important for protein stability (Kuras et al. 1995; de Vitry et al. 2004). HCF153 from *Arabidopsis* is probably involved in assembly of the cytochrome *b*_6_ *f* complex, because mutants exhibit drastically reduced amounts of the major subunits, while their translation is not impaired (Lennartz et al. 2006). However, the molecular function has yet not been revealed.

PSI assembly is initiated by the co-translational insertion of PsaA and PsaB, which then bind to their co-factors to form the PsaAB reaction center. Subsequently, PsaC with its two [4Fe–4S] clusters is attached, followed by PsaD, PsaE and other subunits (Schöttler et al. 2011). The nuclear-encoded subunits PsaK and PsaG apparently bind to PSI after association of the PSI core and LHCI complexes and stabilize this interaction (Ozawa et al. 2010). Besides the true components of PSI that assist in complex assembly, only a small number of auxiliary proteins were identified so far (Schöttler et al. 2011). Two plastid-encoded proteins, Ycf3 and Ycf4, are both essential for PSI accumulation in *Chlamydomonas* (Boudreau et al. 1997). In tobacco, *ycf4* mutants maintain photoautotrophic growth although they are severely affected in their photosynthetic capacity (Krech et al. 2012). In addition, two nuclear-encoded proteins were found to be essential for PSI accumulation in higher plants: a Ycf3-interacting protein (Y3IP1) (Albus et al. 2010) and Pyg7-1 whose function has not been clearly defined yet (Stöckel et al. 2006).

## Conclusion

The chloroplast is a semiautonomous cell organelle of prokaryotic origin embedded into the environment of a eukaryotic cell. In order to allow proper chloroplast development and its adjustment to endo and exogenous stimuli, the nucleus provides a large number of factors that regulate different processes inside the chloroplast (Fig. 1). In this article, we gave an overview of the different steps in chloroplast gene expression and in the assembly of the photosynthetic thylakoid membrane complexes and the ways they are regulated predominantly by nuclear-encoded proteins. Even though more and more of these factors are identified, e.g. through forward genetic screens and mass spectrometric approaches, the precise modes of action are still largely unknown. However, some of these mechanisms are about to be understood, and it becomes more and more clear that chloroplast gene expression as well as complex assembly are highly dynamic processes. We have only begun to understand how light quality and quantity, availability of nutrients, and developmental and stress signals take influence on the processes inside the chloroplast. Uncovering all the different regulatory mechanisms will be an exciting challenge for future research.

## Abbreviations

CES: Control by epistasis of synthesis
HAT: Half-a-TPR
NEP: Nuclear-encoded RNA polymerase
PEP: Plastid-encoded RNA polymerase
PPR: Pentatricopeptide repeat
PSI: Photosystem I
PSII: Photosystem II
SD: Shine–Dalgarno
TPR: Tetratricopeptide repeat
